# Effects of Melatonin and *Akkermansia muciniphila* on the Gut-Liver Axis in a MASLD-Associated Liver Fibrosis Model: An Integrative Multi-Omic Approach

**DOI:** 10.3390/antiox15030306

**Published:** 2026-02-28

**Authors:** Alba González-Robles, Beatriz San Miguel, Sara Román-Sagüillo, María Juárez-Fernández, José L. Mauriz, Susana Martínez-Flórez, Esther Nistal, María Victoria García-Mediavilla, Sonia Sánchez-Campos

**Affiliations:** Instituto Universitario de Biomedicina (IBIOMED), Universidad de León, 24007 León, Spain; algonr@unileon.es (A.G.-R.); bsanv@unileon.es (B.S.M.); sroms@unileon.es (S.R.-S.); mjuarf@unileon.es (M.J.-F.); jl.mauriz@unileon.es (J.L.M.); smarf@unileon.es (S.M.-F.); menisg@unileon.es (E.N.); ssanc@unileon.es (S.S.-C.)

**Keywords:** gut microbiota, *Akkermansia muciniphila*, melatonin, liver fibrosis, MASLD

## Abstract

Metabolic dysfunction-associated steatotic liver disease (MASLD) is the most common cause of chronic liver disease worldwide. Fibrosis is the main prognostic factor and the last reversible stage before cirrhosis, yet therapeutic options remain limited. Given the strong contribution of gut dysbiosis to MASLD progression, strategies targeting the gut microbiota are of growing interest. This study aims to evaluate the effect of melatonin, a well-known antioxidant, anti-inflammatory and antifibrotic compound, and *Akkermansia muciniphila*, a next-generation probiotic, on an MASLD-associated liver fibrosis model. Eight-week-old C57BL/6J mice were fed a control or Western diet supplemented with fructose and intraperitoneal CCl_4_ to induce liver fibrosis. After eight weeks, the animals received either no intervention, melatonin, *A. muciniphila*, or both for four weeks. Serum biochemistry, liver histology and gut and liver gene expression were evaluated and multi-omic analyses were performed, including gut microbiota profiling and faecal metabolomics. Statistical analyses assessed intergroup differences and correlations across datasets. Both interventions partially restored gut microbiota composition and functionality and modulated hepatic and intestinal gene expression. Melatonin and *A. muciniphila* exerted protective effects against MASLD-associated fibrosis, which supports their potential as adjunctive therapeutic strategies to mitigate liver injury through modulation of the gut–liver axis.

## 1. Introduction

Metabolic dysfunction-associated steatotic liver disease (MASLD) is the leading cause of chronic liver disease worldwide and currently affects 30% of the adult population [1]. Its prevalence has risen substantially over the last three decades, with an expected exponential increase in accordance with the escalating rates of obesity and metabolic disorders [2]. The pathogenesis of MASLD follows the “multiple hit hypothesis”, in which genetically susceptible individuals are affected by environmental and lifestyle-related factors, particularly sedentarism and hypercaloric diets rich in fat and refined sugars, that lead to insulin resistance, obesity, chronic low-grade inflammation and gut microbiota dysbiosis [3]. MASLD encompasses a wide spectrum of liver injuries, including simple steatosis, steatohepatitis, fibrosis and cirrhosis [4]. Liver fibrosis is the result of chronic inflammation that triggers excessive accumulation of extracellular matrix, leading to structural alteration and functional impairment [5]. The presence and grade of liver fibrosis are the most important prognostic determinants in MASLD. Moreover, liver fibrosis is the last reversible stage before cirrhosis, and hence its clinical relevance [6,7].

Gut microbiota acts as a metabolically active organ [8] that plays a crucial role in maintaining host health as it performs a wide range of essential functions that contribute to human physiology [9]. Many extrinsic factors can affect gut microbiota, leading to an imbalance known as dysbiosis, which involves compositional and functional changes [10]. MASLD is linked to dysbiosis, given the bidirectional crosstalk between gut and liver through the gut–liver axis [11]. Dietary patterns and metabolic disturbances characteristic of MASLD alter gut microbial communities, and at the same time, dysbiosis contributes to gut barrier disruption and increased intestinal permeability, enabling bacteria and their components to reach the liver, where they activate inflammatory pathways and stimulate innate immune responses that aggravate MASLD development. Gut dysbiosis also contributes to disease progression by increasing energy yield from diet, modulating bile acid metabolism and enhancing endogenous ethanol production [8,10,11].

To date, lifestyle interventions, including diet and exercise, remain the cornerstone of MASLD management, as pharmacological treatments remain limited and restricted to specific patient subgroups. Although lifestyle interventions have proven effective, their major drawback is the difficulty of sustaining these changes in the long term [12,13]. Given the critical role of gut dysbiosis in MASLD progression, modulation of gut microbiota through various strategies, such as the administration of prebiotics, probiotics and synbiotics, constitutes a promising therapeutic approach [14]. In this context, and beyond its well-established immunomodulatory, anti-inflammatory and antifibrotic properties [15,16], melatonin attenuates oxidative stress by increasing antioxidant enzyme activities, enhancing total antioxidant capacity and reducing lipid peroxidation [17]. In parallel, melatonin acts as a prebiotic by reversing gut dysbiosis and enhancing intestinal barrier integrity [18]. These effects, combined with its proven ability to reduce body weight gain, improve hepatic steatosis, reduce inflammation and enhance insulin sensitivity, underscore melatonin as a potential candidate for MASLD management [19]. Likewise, *Akkermansia muciniphila*, a next-generation probiotic, has been introduced as a promising therapeutic option for MASLD, thanks to its capacity to improve insulin sensitivity and maintain gut homeostasis and gut barrier functionality, alleviating hepatic steatosis and inflammation [14,20].

This study aims to evaluate the combined effect of melatonin and *A. muciniphila* administration on metabolic parameters, the severity of the hepatic disease, the gut barrier integrity and the gut microbiota composition and functionality in an in vivo model of MASLD-associated liver fibrosis.

## 2. Materials and Methods

### 2.1. Experimental Design

All procedures were performed in accordance with the European Research Council guidelines for the care and use of laboratory animals and were approved by the Institutional Animal Care and Use Committee of the University of León (OEBA-ULE-004-2021), followed by authorisation from the competent authority, the Junta de Castilla y León.

#### 2.1.1. Animal Model

**Figure 1:**
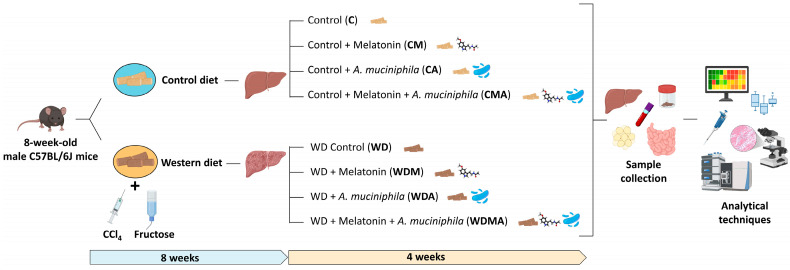
Experimental design: 8-week-old C57BL/6J mice were fed either a Control or Western Diet for 8 weeks. WD-fed mice received fructose-supplemented drinking water and intraperitoneal injections of CCl_4_. Then, both groups were split into four subgroups based on diet alone (C and WD groups), melatonin administration (CM and WDM groups), *Akkermansia muciniphila* administration (CA and WDA groups) and combined melatonin and *A. muciniphila* administration for 4 weeks.

Eight-week-old male C57BL/6J mice were divided into two groups based on their diet: Control (C) (18% of energy from fat; 114 Teklad Global #2018 Diet, Harlan Laboratories) (*n* = 40) and Western Diet (WD) (43% of energy from fat and 2% cholesterol; TD.160279; Envigo Teklad Diets, Madison, WI, USA) (*n* = 40). This second group received fructose-supplemented drinking water (150 mg mL^−1^) and an intraperitoneal injection of CCl_4_ (0.5 mL kg^−1^) at weeks 1, 2, 4 and 6. The C group received intraperitoneal injections of the vehicle of CCl_4_ (corn oil) at the same intervals. After 8 weeks, both groups (C and WD) were split into four subgroups based on diet solely (C [*n* = 8] and WD [*n* = 12] groups), melatonin administration (CM [*n* = 8] and WDM [*n* = 12] groups), *Akkermansia muciniphila* administration (CA [*n* = 8] and WDA [*n* = 12] groups) and combined melatonin and *A. muciniphila* administration (CMA [*n* = 8] and WDMA [*n* = 12] groups) (Figure 1). The mice were housed under controlled conditions of temperature, humidity and lighting, and had free access to water and food, which they consumed *ad libitum*. Body weight, food and water intake were monitored weekly. At the 12th week, the animals were euthanised by cardiac puncture under anaesthesia and blood, liver, ileum, adipose tissue and faecal and caecal contents were collected.

#### 2.1.2. Bacterial Strain and Growth Conditions

*Akkermansia muciniphila* (CIP 107961^T^) was cultured in brain heart infusion medium (OXOID Ltd., Basingstoke, Hampshire, UK) at 37 °C for 48 h under anaerobic conditions, as previously described [21]. Cultures were centrifuged at 9000 rpm for 10 min, washed twice with sterile phosphate-buffered saline (PBS, pH 7.2), and resuspended in 200 μL of 10% skim milk in PBS supplemented with 3% cysteine to a final concentration of 2 × 10^8^ CFU for preservation. The suspension was stored at −80 °C and, prior to administration, was centrifuged again and resuspended in PBS, maintaining the same bacterial concentration. All procedures were carried out under strictly anaerobic conditions.

#### 2.1.3. Dosage Information

Melatonin was dissolved in absolute ethanol, and further dilutions were prepared in 0.9% NaCl solution until a final ethanol concentration of 2.5% was reached. Melatonin was administered intraperitoneally in the evenings at a dose of 20 mg kg^−1^ day^−1^, a dosage selected based on previous studies from our group in which this regimen was experimentally optimised [22]. Groups not supplemented with melatonin received daily injections of the vehicle (2.5% ethanol in 0.9% NaCl solution) alone.

*Akkermansia muciniphila* was administered five times per week by oral gavage at a dose of 2 × 10^8^ CFU in 200 μL of PBS as the vehicle, established according to previous work from our group [23]. Groups not supplemented with *A. muciniphila* received the vehicle alone.

### 2.2. Sample Collection

Blood samples were collected at the 8th week and at the end of the experimental period, and serum was immediately isolated for subsequent biochemical analysis. Liver and adipose tissue were harvested and weighed. Faecal and caecal contents were collected for gut microbiota compositional and metabolomic analyses. The left lobe of the liver and the ileum were immersed in RNA*later*™ stabilisation solution (AM7021; Invitrogen, Thermo Fisher Scientific Inc.; Waltham, MA, USA) for 24 h and then stored at −80 °C for subsequent gene expression analyses. The right lobe of the liver was fixed in 10% formalin for histological evaluation.

### 2.3. Biochemical Analysis

Alanine aminotransferase (ALT), aspartate aminotransferase (AST) and gamma-glutamyl transferase (GGT) activities, as well as glucose, triglycerides and cholesterol levels, were determined in serum samples by the Instrumental Techniques Laboratory of the University of León using standard procedures.

### 2.4. Histopathology

Paraffin-embedded liver samples were sectioned and stained with haematoxylin and eosin (H&E). Histological assessments were performed by two expert examiners blinded to the experimental design. The extent of hepatic lesions was evaluated according to the NAFLD activity score (NAS) to provide a numerical score based on three histological features: steatosis (0–3), lobular inflammation (0–3) and hepatocellular ballooning (0–2) [24]. Liver fibrosis was assessed using Masson’s trichrome staining and a semiquantitative scoring system provided by a histopathology service from the University of León, ranging from 0 to 10, where 0 indicates absence of fibrosis and 10 corresponds to extreme fibrosis.

### 2.5. Gene Expression Analysis

#### 2.5.1. RNA Extraction

For total RNA isolation, approximately 50 mg of each sample was homogenised in 1 mL of TRIzol™ reagent (15596026; Invitrogen, Thermo Fisher Scientific Inc., Waltham, MA, USA), following the manufacturer’s protocol, which is based on phase separation with chloroform, RNA precipitation with isopropanol and subsequent washing and resuspension. The concentration of total RNA was measured with a NanoDrop 1000 spectrophotometer (Thermo Fisher Scientific Inc.; Waltham, MA, USA).

#### 2.5.2. Quantitative Real-Time PCR (RT-qPCR)

For the reverse transcription reaction, first-strand cDNA was synthesised using the High-Capacity cDNA Reverse Transcription Kit (4368814; Thermo Fisher Scientific Inc., Waltham, MA, USA). Relative gene expression assays were carried out using the Applied Biosystems QuantStudio 5 Real-Time PCR System (Thermo Fisher Scientific Inc., Waltham, MA, USA) and commercially available TaqMan^®^ Gene Expression Assays (Applied Biosystems, Thermo Fisher Scientific Inc., Waltham, MA, USA) (Table S1). Glyceraldehyde-3-phosphate dehydrogenase (*Gapdh*) was used as internal control, and the 2^−∆∆Ct^ method was applied to determine relative changes in gene expression levels.

### 2.6. Gut Microbiota Compositional Analysis

#### 2.6.1. Faecal DNA Extraction

Genomic DNA from faecal samples was extracted using the QIAamp Fast DNA Stool Mini Kit (51604; Qiagen, Hilden, Germany) according to the manufacturer’s instructions, with some modifications, as previously described [25]. Briefly, an initial bead-beating step using PowerBead Pro Tubes (Qiagen, Hilden, Germany) was included, and the lysis temperature was increased to 95 °C to aid in the recovery of DNA from bacteria that are difficult to lyse. DNA concentration was determined using a NanoDrop 1000 spectrophotometer (Thermo Fisher Scientific Inc., Waltham, MA, USA), and DNA samples were stored at −20 °C until further analysis.

#### 2.6.2. Amplification, Sequencing of 16S rRNA and Bioinformatic Analysis

Amplification of the 16S rRNA V3-V4 hypervariable region was performed by PCR, as previously described [26]. PCR assays were carried out in triplicate, and their products were pooled and purified using the Wizard^®^ Genomic DNA Purification Kit (Promega, Madison, WI, USA).

Gut microbiota compositional analysis was carried out using the Illumina MiSeq platform, according to the manufacturer’s instructions, using the MiSeq Reagent Kit v3 and 300 bp paired-end reads. The Illumina bcl2fastq^©^ program was used to demultiplex the sequencing data, and FastQC software (version 0.11.9) was used for post-sequencing quality control [27].

Analysis of the generated raw sequence data was performed using the Quantitative Insights into Microbial Ecology (QIIME 2) software (version 2022.2) [28] and the DADA2 plugin [29], including the joining of forward and reverse reads, chimera removal, data filtering, and taxonomic annotation. Reads were organised into operational taxonomic units (OTUs). A fitted classifier trained on the SILVA database (release 138 QIIME) was used for taxonomic assignment, with a clustering threshold of 99% similarity. Only OTUs containing at least 10 sequence reads were considered significant. Genus-level abundance tables were further filtered using MicrobiomeAnalyst [30] prior to downstream statistical analyses. Filtered data were subsequently normalised manually to relative abundances (sum = 1).

### 2.7. Faecal Metabolomic Analysis

Faecal metabolome was determined by liquid chromatography–mass spectrometry (LC-MS) at MS-Omics (Copenhagen, Denmark). Approximately 50–100 mg of faecal content was used for metabolite extraction. For each sample, 2 µL of eluent solution per mg of faeces was added, followed by vortex mixing and centrifugation. The resulting supernatants were transferred to centrifugation filter tubes, and the filtrate was diluted 10-fold in eluent solution prior to LC-MS analysis. For quality control, a pooled sample was prepared by combining small aliquots from each individual sample.

The LC-MS method was performed using a UPLC system (Vanquish; Thermo Fisher Scientific Inc., Waltham, MA, USA) coupled with a high-resolution quadrupole-orbitrap mass spectrometer (Q Exactive™ HF Hybrid Quadrupole-Orbitrap; Thermo Fisher Scientific Inc., Waltham, MA, USA). An electrospray ionisation interface was used as the ion source. Analysis was conducted in negative and positive ionisation modes. Our quality control sample was analysed in MS/MS mode for compound identification. Data were processed using Compound Discoverer 3.2 (ThermoFisher Scientific Inc., Waltham, MA, USA). Metabolomic datasets were subsequently pre-processed and filtered using MetaboAnalyst [31] prior to statistical and correlation analyses, including quality control and feature filtering.

### 2.8. Statistical Analysis

Data are expressed as the mean ± standard deviation (SD). Significant differences between two groups (8th week) were evaluated by Student’s *t*-test, while comparisons among multiple experimental groups (12th week) were performed using one-way analysis of variance (ANOVA), followed by pairwise Student’s *t*-tests and Fisher’s LSD post hoc test when applicable.

Statistical differences in gut microbiota composition and metabolomic data were assessed by a non-parametric Kruskal–Wallis test, followed by pairwise Mann–Whitney U tests when *p* < 0.05; Dunn’s test was applied for correction for multiple testing. Permutational multivariate analysis of variance (PERMANOVA) was used to assess the contribution of different factors to bacterial community distribution. Analyses were conducted using R software version 4.3.2 (R-project, Vienna, Austria) and GraphPad Prism 8 (San Diego, CA, USA).

#### Correlation Analysis

Correlation analyses were performed to explore associations between gut microbiota composition, faecal metabolomic profiles, serum biochemical parameters and hepatic and intestinal gene expression.

Spearman’s rank correlation coefficients were calculated due to the non-parametric distribution of the data, using the *associate* function from the R package *microbiome*. *p*-Values were adjusted for multiple testing using the Benjamini–Hochberg false discovery rate (FDR) method. Correlations with adjusted q-values < 0.05 were considered statistically significant. Correlation matrices were visualised as heatmaps using the *heat* function of the *microbiome* package. All analyses were conducted in R software (R-project, Vienna, Austria).

## 3. Results

### 3.1. Western Diet Induces Metabolic and Histological Alterations and Hepatic and Intestinal Gene Expression Deregulation

After 8 weeks, the group that received WD feeding developed multiple alterations consistent with MASLD. Biochemical analyses revealed significant increases in ALT (+54.6%) and AST (+42.4%) levels in comparison with the control diet-fed group (Table S2), indicating hepatocellular damage. Fasting glucose and total cholesterol concentrations were also markedly elevated (+20.5% and +133.5%, respectively) (Table S2), reflecting the onset of metabolic dysfunction and a possible early impairment of insulin sensitivity.

**Figure 2:**
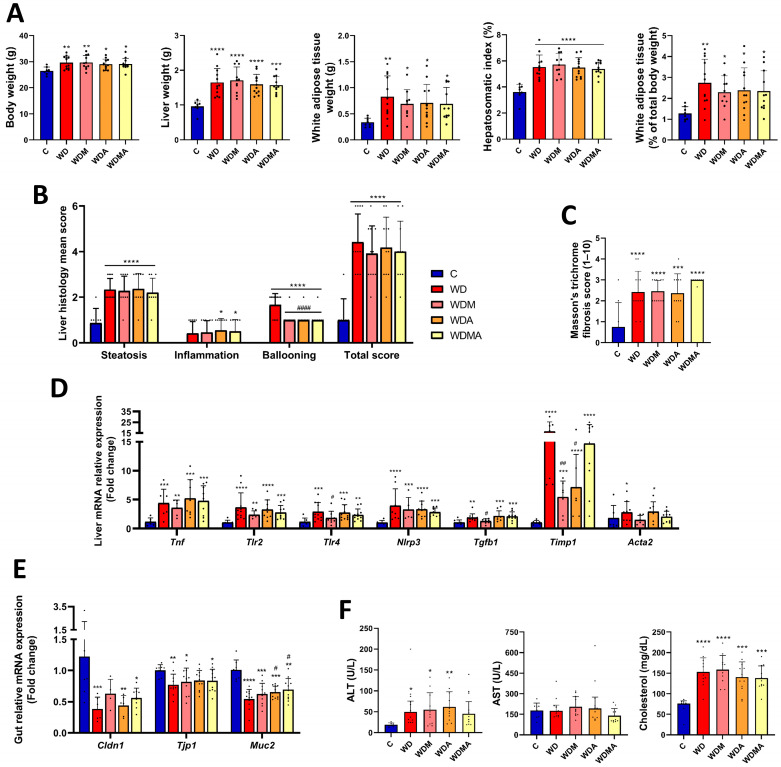
Effect of Western Diet, melatonin and *A. muciniphila* administration on MASLD-associated features development. (**A**) Body, liver and white adipose tissue weights and hepatosomatic index (liver weight/body weight × 100). (**B**) NAFLD activity score (NAS) calculated from individual scores for steatosis, lobular inflammation and hepatocellular ballooning. (**C**) Fibrosis evaluation on Masson’s trichrome-stained sections using a semiquantitative scoring system ranging from 1 to 10, as provided by the histopathology service. (**D**) Relative mRNA expression in liver tissue of genes involved in inflammation (*Tnf*, *Tlr2*, *Tlr4*, *Nlrp3*, *Tgfb1*) and fibrosis (*Timp1*, *Acta2*). (**E**) Relative mRNA expression in gut tissue of genes involved in intestinal barrier function (*Cldn1*, *Tjp1*, *Muc2*). (**F**) ALT and AST levels and cholesterol concentration in serum. At least *n* = 5 were used in each experimental group. * *p* < 0.05 vs. C; ** *p* < 0.01 vs. C; *** *p* < 0.001 vs. C; **** *p* < 0.0001 vs. C; ^#^ *p* < 0.05 vs. WD; ^##^ *p* < 0.01 vs. WD; ^####^ *p* < 0.0001 vs. WD.

At the 12th week, at the end of the experimental period, the WD group, which did not receive any treatment, showed significantly higher body, liver, and white adipose tissue weights (+14.2%, +72.7% and +146.7%, respectively) and an increased hepatosomatic index (Figure 2A). These changes reflect increased adiposity and marked liver enlargement associated with lipid accumulation, with white adipose tissue expansion being evident both in absolute terms and relative to total body weight. Histological examination revealed increased steatosis, hepatocellular ballooning and total NAS score with respect to the control group (Figure 2B and Figure S1), along with the presence of liver fibrosis (Figure 2C and Figure S2). At the molecular level (Figure 2D), WD-fed mice exhibited upregulation of hepatic pro-inflammatory (*Tnf*, *Tlr2*, *Tlr4*, *Nlrp3*) and profibrogenic (*Timp1*, *Acta2*) genes, consistent with low-grade inflammation and early fibrogenic remodelling. Moreover, intestinal barrier integrity was compromised, as shown by the significant downregulation of genes encoding key tight junction proteins (*Cldn1*, *Tjp1*) and mucosal barrier formation (*Muc2*) (Figure 2E), indicative of impaired epithelial integrity and reduced mucosal protection.

### 3.2. Melatonin and A. muciniphila Exert Limited Biochemical and Histological Effects but Induce Hepatic and Intestinal Gene Expression Changes

Melatonin and *A. muciniphila* administration to the control diet-fed mice did not modify any of the biochemical, morphometric or histological analysed parameters. As no differences were observed among control groups (Figure S3), only the untreated control group (C) was used for subsequent analyses. In WD-fed mice, interventions showed partial improvements in different outcomes. Both the WDA and WDMA groups displayed a trend towards lower serum cholesterol concentrations, while the WDMA group showed a modest, non-significant reduction in ALT and AST levels (Figure 2F), suggesting a possible initial improvement in liver damage. Liver weight tended to decrease in response to *A. muciniphila* administration, whereas white adipose tissue weight, both in absolute terms and expressed as a percentage of the total body weight, was slightly but non-significantly reduced across all intervention groups (Figure 2A). Histologically, hepatocellular ballooning was significantly reduced in the WDM, WDA and WDMA groups, although the total NAS score remained unchanged (Figure 2B).

At the molecular level, melatonin treatment significantly decreased hepatic expression of *Tlr4*, *Tgfb1* and *Timp1*, and showed a trend towards reduced expression of *Tnf*, *Tlr2*, *Nlrp3* and *Acta2* (Figure 2D). At the intestinal level, melatonin showed a trend towards increased *Cldn1* expression, suggesting a partial restoration of tight junction integrity (Figure 2E). *A. muciniphila* administration significantly reduced hepatic *Timp1* expression (Figure 2D), potentially attenuating profibrogenic activity, and significantly increased intestinal *Muc2* expression (Figure 2E), supporting mucosal barrier function. Finally, the combined treatment tended to decrease hepatic *Tlr2*, *Tlr4* and *Nlrp3* expression (Figure 2D), indicating a tendency to reduced hepatic inflammatory and profibrogenic signalling, and was associated with a significant upregulation of *Muc2*, together with a trend towards increased intestinal *Cldn1* expression (Figure 2E), which may collectively contribute to improving intestinal barrier integrity.

Overall, these findings indicate that melatonin and *A. muciniphila*, alone or in combination, partially modulate hepatic and intestinal molecular pathways and histological features, reflecting a modest improvement in liver function and gut barrier-related parameters in WD-fed mice.

### 3.3. Melatonin and A. muciniphila Modulate Gut Microbiota Diversity and Composition After WD-Induced Dysbiosis

**Figure 3:**
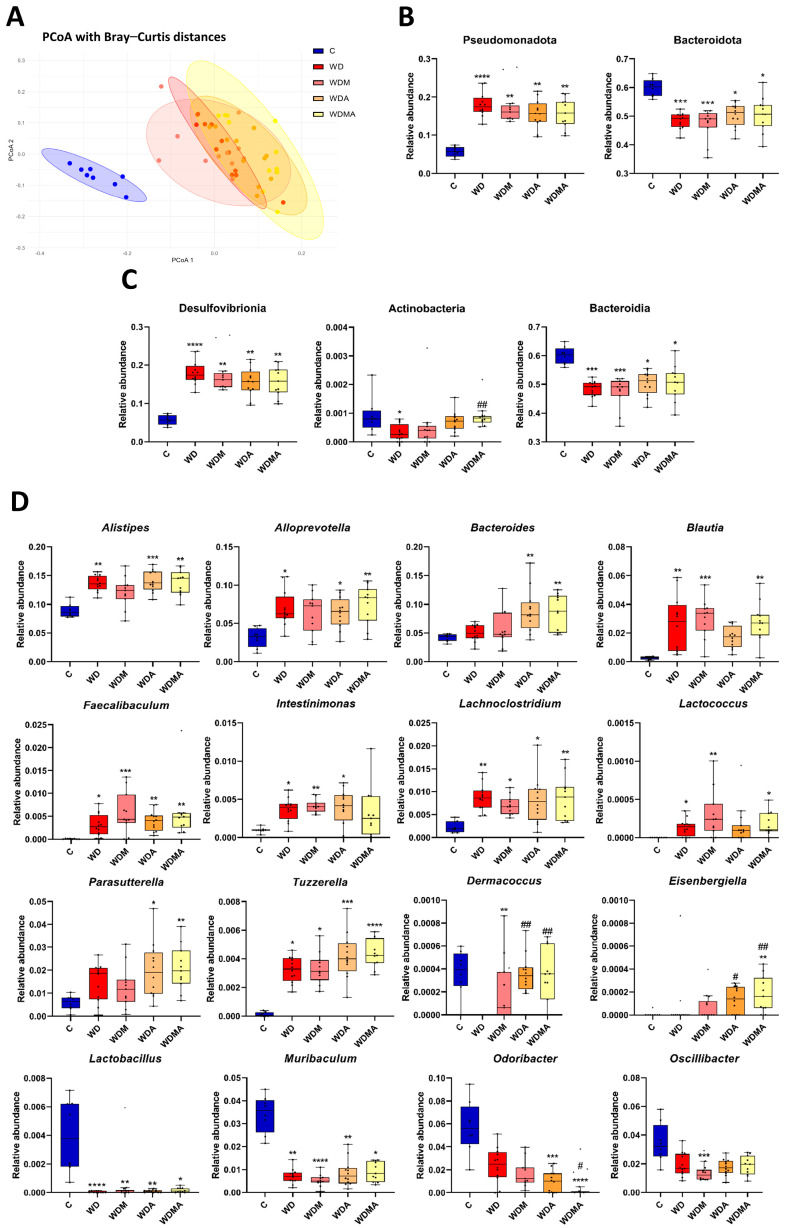
Effect of Western Diet, melatonin and *A. muciniphila* administration on gut microbiota diversity and composition. (**A**) Principal coordinates analysis (PCoA) plot based on the Bray–Curtis dissimilarity index at the genus level, representing β-diversity. (**B**) Relative abundance in the different experimental groups at the phylum level, (**C**) class level and (**D**) genus level. At least *n* = 7 were used in each experimental group. * *p* < 0.05 vs. C; ** *p* < 0.01 vs. C; *** *p* < 0.001 vs. C; **** *p* < 0.0001 vs. C; ^#^ *p* < 0.05 vs. WD; ^##^ *p* < 0.01 vs. WD.

To assess global changes in gut microbial community structure, α and β-diversity analyses were performed. For α-diversity (Figure S4), the Simpson index showed a significant overall difference across groups (Kruskal–Wallis *p* = 0.043), although no significant pairwise comparisons were detected. In contrast, the Shannon index did not show significant overall differences (Kruskal–Wallis *p* = 0.074). β-diversity analysis (Figure 3A) based on Bray–Curtis dissimilarity and Principal Coordinates Analysis (PCoA) revealed a clear group-dependent clustering, primarily driven by the dietary intervention, as confirmed by PERMANOVA (*p* = 0.001), with the group factor explaining 66.67% of the total variance (R^2^ = 0.6667).

The fibrosis-inducing protocol produced marked alterations in gut microbiota composition at multiple taxonomic levels. At the phylum level, WD-fed mice showed a significant increase in the relative abundance of Pseudomonadota, accompanied by a significant reduction in Bacteroidota (Figure 3B). At the class level, Desulfovibrionia was significantly enriched, whereas Actinobacteria and Bacteroidia were significantly reduced (Figure 3C).

At the genus level, the WD group significantly increased the relative abundance of *Alistipes*, *Alloprevotella*, *Blautia*, *Faecalibaculum*, *Intestinimonas*, *Lachnoclostridium*, *Lactococcus* and *Tuzzerella*. Conversely, *Dermacoccus*, *Lactobacillus* and *Muribaculum* were significantly reduced (Figure 3D).

Both melatonin and *A. muciniphila* showed modulatory effects at the genus level (Figure 3D). *Alistipes* abundance decreased in WDM compared to the WD group, approaching control values, and *Blautia* was reduced in WDA. However, intervention effects were most evident in the WDMA group, where the class Actinobacteria was significantly increased, reaching values comparable to controls (Figure 3C). At the genus level, *Dermacoccus* significantly increased in WDA and WDMA, accompanied by a similar trend in *Eisenbergiella*. *Muribaculum* showed a non-significant increase in WDMA, while *Odoribacter* was significantly reduced in this group compared to WD (Figure 3D).

Together, these results indicate that Western diet feeding is the main determinant of gut microbial community structure in this model. While α-diversity analyses showed only subtle overall differences, taxonomic-level analyses reveal that the interventions have already induced genus-specific shifts, particularly under the combined treatment, suggesting that the gut microbiota composition and structure were modulated, partially counteracting diet-related alterations.

### 3.4. Melatonin and A. muciniphila Partially Restore WD-Impaired Gut Microbiota Functionality

**Figure 4:**
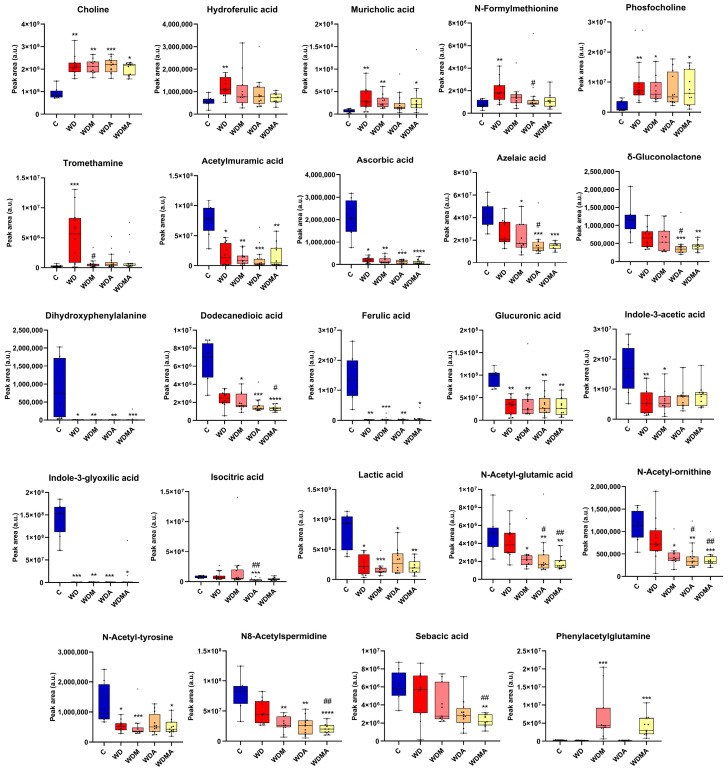
Effect of Western diet, melatonin and *A. muciniphila* on the faecal metabolome. At least *n* = 8 were used in each experimental group. * *p* < 0.05 vs. C; ** *p* < 0.01 vs. C; *** *p* < 0.001 vs. C; **** *p* < 0.0001 vs. C; ^#^ *p* < 0.05 vs. WD; ^##^ *p* < 0.01 vs. WD vs. WD.

The fibrogenic intervention not only altered microbiota composition but also markedly disrupted its functional profile, as reflected by shifts in faecal metabolite concentrations. WD feeding significantly increased several metabolites (choline, hydroferulic acid, muricholic acid, N-formylmethionine, phosphocholine and tromethamine) while reducing numerous compounds linked to microbial metabolism and host–microbiota interactions (acetylmuramic acid, ascorbic acid, dihydroxyphenylalanine, ferulic acid, glucuronic acid, indole-3-acetic acid, indole-3-glyoxylic acid, lactic acid and N-acetyl-tyrosine) (Figure 4). These changes indicate possible functional microbial alterations accompanying the compositional shifts.

Administration of *A. muciniphila* and/or melatonin partially reversed WD-induced metabolomic disturbances, generally lowering metabolites elevated in WD mice towards control levels, with significant effects for N-formylmethionine, following *A. muciniphila* administration, and tromethamine, in response to melatonin. Interestingly, certain metabolites (azelaic acid, δ-gluconolactone, dodecanedioic acid, isocitric acid, N-acetyl-glutamic acid, N-acetyl-ornithine, N8-acetylspermidine and sebacic acid) decreased further relative to WD, diverging from the control profile. In contrast, phenylacetylglutamine increased significantly in the WDM and WDMA groups (Figure 4).

### 3.5. Correlation Analyses Reveal Interactions Between Host Biochemical and Molecular Markers, Gut Microbiota Composition and Faecal Metabolomic Profiles

**Figure 5:**
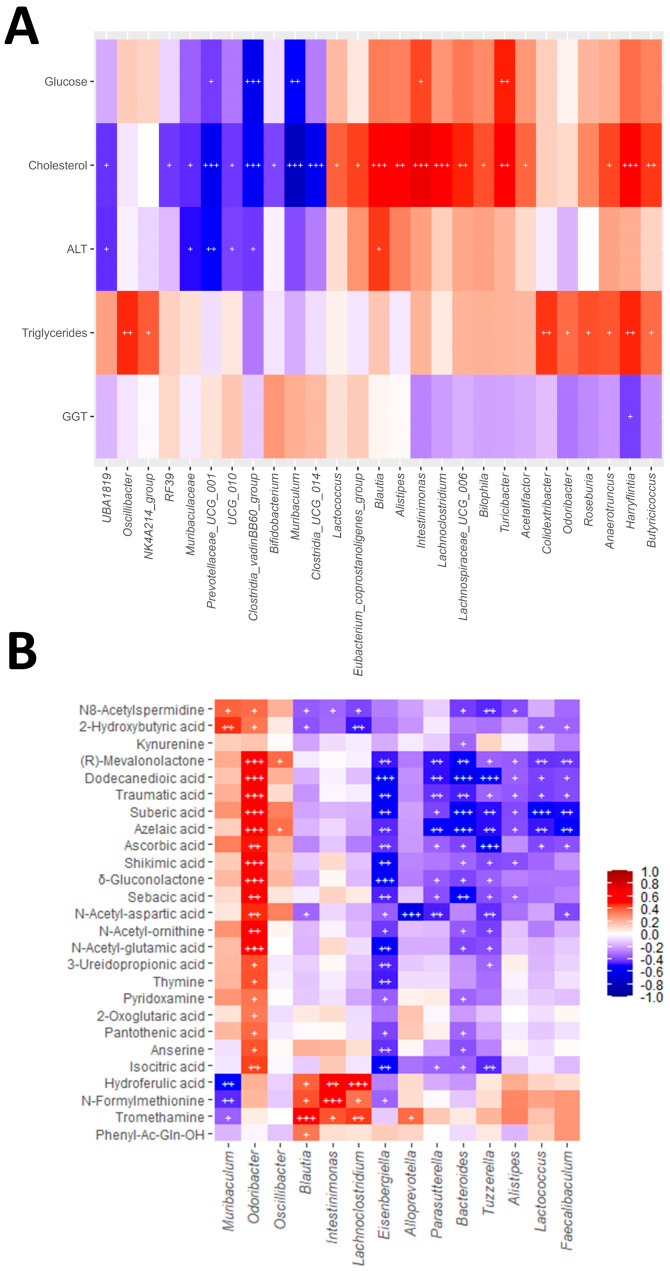
Correlation analysis. Heatmaps showing Spearman correlations between (**A**) serum biochemical parameters and bacterial genera and (**B**) faecal concentrations of metabolites and bacterial genera. Each square represents the Spearman correlation coefficient (q < 0.05). Red and blue cells represent positive and negative correlations, respectively. White crosses designate the level of significance: ^+^ q < 0.05; ^++^ q < 0.01; ^+++^ q < 0.001.

Correlation analyses revealed consistent associations between gut microbial taxa, faecal metabolites and host biochemical parameters and hepatic and intestinal gene expression (Figure 5 and Figure S5). Among the genera altered by WD, *Alistipes*, *Blautia*, *Intestinimonas* and *Lachnoclostridium* showed positive correlations with serum cholesterol (Figure 5A), supporting their possible association with WD-induced dyslipidaemia. *Alistipes* and *Blautia*, additionally, correlated positively with hepatic expression of pro-inflammatory and profibrogenic genes (Figure S5B), and *Blautia* also correlated with ALT levels (Figure 5A), possibly linking these taxa to liver injury and fibrogenic responses. *Intestinimonas* was also associated with fasting glucose (Figure 5A), suggesting a contribution to metabolic alterations. In contrast, *Muribaculum* abundance correlated negatively with glucose and cholesterol concentrations (Figure 5A) and positively with the expression of intestinal barrier integrity-related genes (Figure S5D), highlighting a potential protective role. Similarly, *Lactobacillus* was inversely associated with hepatic inflammatory and fibrogenic markers (Figure S5B), suggesting a role in ameliorating liver injury.

Several metabolites that changed significantly in response to WD and melatonin and/or *A. muciniphila* were also linked to host biochemical and molecular parameters. N-formylmethionine and hydroferulic acid correlated positively with serum cholesterol (Figure S5A), and N-formylmethionine also correlated with hepatic *Timp1* expression (Figure S5C). Tromethamine was likewise associated with *Timp1*, *Tlr4* and *Tnf* (Figure S5C), pointing to potential involvement in pro-inflammatory signalling. Conversely, dicarboxylic acids, including sebacic and dodecanedioic acids, showed negative correlations with hepatic inflammatory and fibrogenic markers (Figure S5C). A similar pattern was observed for δ-gluconolactone, N-acetyl-ornithine, N-acetyl-glutamic acid, N8-acetylspermidine and ascorbic acid, whose depletion was consistently linked to enhanced liver injury (Figure S5C).

Genus–metabolite correlations (Figure 5B) revealed consistent associations between bacterial taxa and specific metabolites. *Blautia*, *Intestinimonas* and *Lachnoclostridium* correlated positively with N-formylmethionine, hydroferulic acid and tromethamine, whereas *Muribaculum* displayed opposite associations with these metabolites, in line with its suggested protective role. In addition, several taxa were connected to dicarboxylic acids: *Odoribacter* correlated positively with dodecanedioic, azelaic and sebacic acids and also showed positive associations with other metabolites such as δ-gluconolactone, N-acetyl-glutamic acid, N-acetyl-ornithine, ascorbic acid and isocitric acid. Conversely, *Bacteroides*, *Eisenbergiella*, *Faecalibaculum*, *Lactococcus*, *Parasutterella* and *Tuzzerella* were consistently negatively associated with dicarboxylic acids. Altogether, these associations illustrate coordinated networks linking microbial shifts, metabolite profiles and host responses in WD-induced metabolic associated liver disease, partially reshaped by melatonin and *A. muciniphila* treatment.

## 4. Discussion

MASLD is characterised by hepatic lipid accumulation driven by hypercaloric diets rich in sugars and saturated fats, typically referred to as “Western Diet” [32,33]. Patients frequently display gut dysbiosis and impaired intestinal barrier integrity, underscoring the role of the gut–liver axis in disease development [11]. Lifestyle interventions remain the cornerstone of MASLD management; however, long-term adherence is limited [34]. Given the key role of gut dysbiosis in disease progression, modulation of the gut microbiota using prebiotics and probiotics has emerged as a promising therapeutic approach [10,11].

Melatonin, a well-known antioxidant and anti-inflammatory molecule, has been shown to enhance endogenous antioxidant defences and attenuate lipid peroxidation, thereby limiting oxidative stress-driven tissue damage [35]. Additionally, melatonin has demonstrated prebiotic properties, counteracting diet-induced dysbiosis and metabolic alterations [36,37,38]. Likewise, *Akkermansia muciniphila*, considered a next-generation probiotic, has shown protective effects against obesity, insulin resistance, steatosis and fibrosis, through its impact on gut microbiota composition [23,39].

This study aims to evaluate the effects of melatonin and *A. muciniphila*, administered either alone or in combination, on metabolic parameters, liver disease severity, gut barrier integrity and gut microbiota composition and functionality in an in vivo model of MASLD-associated liver fibrosis.

Overall, melatonin and/or *A. muciniphila* administration induced only modest improvements in hepatic and systemic parameters in this MASLD-associated liver fibrosis model. Although some trends were observed, such as lower serum cholesterol levels and liver weight in *A. muciniphila*-treated groups and subtle reductions in transaminases in the WDMA group, these effects did not translate into consistent or robust improvements across biochemical, histological or fibrotic endpoints. This contrasts with previous studies reporting more pronounced metabolic and hepatic benefits of melatonin [40,41] or *A. muciniphila* supplementation [23,39,42,43], suggesting that, in our experimental setting, maintaining Western diet feeding, together with the relatively short intervention period (four weeks), may have limited the extent of hepatic recovery once liver damage was already established.

At the histological level, hepatocellular ballooning was the only feature consistently ameliorated by treatment. This observation is in line with previous studies reporting improvements in ballooning in response to melatonin [44,45] and describing a negative association between Verrucomicrobiales abundance and ballooning in NASH patients [46] and suggests that melatonin and *A. muciniphila* may preferentially influence specific components of hepatocellular injury rather than advanced structural damage. In parallel, the downregulation of profibrogenic and inflammatory genes, such as *Timp1*, *Tlr4* or *Tgfb1*, in response to melatonin and/or *A. muciniphila* aligns with previous studies [39,47,48] and may reflect early molecular adaptations that were insufficient to elicit detectable histological improvement within the timeframe of this study. In this context, it is worth noting that *A. muciniphila* has also been reported to directly suppress activated hepatic stellate cells in vitro, independently of microbiota modulation [39]. Although this mechanism was not specifically addressed in the present study, our data mainly support an indirect, gut-mediated contribution to fibrosis attenuation, suggesting that multiple, non-mutually exclusive pathways may underlie the observed effects. Together, these findings support the idea that, while short-term intervention can induce molecular and cellular changes, longer or earlier treatments, and potentially optimised dosing or combination strategies, may be required to achieve overt hepatic protection in MASLD under sustained dietary challenge. Notably, the differential responses observed between single and combined treatments, such as the suppression of *Timp1* expression by melatonin or *A. muciniphila* alone but not under combined administration, contrast with the enhanced modulation of specific bacterial taxa observed under combined treatment. This apparent lack of additive or synergistic effects at the molecular level suggests that both interventions engage complex and partially overlapping host pathways. Beyond microbiota modulation, melatonin exerts pleiotropic antioxidant, anti-inflammatory and metabolic actions, while *A. muciniphila* influences host signalling, mucus dynamics and immune responses, which may interact in a context-dependent manner under sustained dietary and toxic challenge.

In contrast to the limited hepatic effects, intestinal readouts appeared more responsive to the interventions. Melatonin tended to restore *Cldn1* expression, consistent with its previously reported ability to upregulate genes involved in epithelial barrier maintenance [49,50], while *A. muciniphila* significantly increased *Muc2* expression and showed a trend towards increased *Tjp1*, in line with its capacity to reinforce mucus production and barrier-related mechanisms [42,51]. These differences between intestinal and hepatic responses suggest that, in our model, melatonin and *A. muciniphila* had already elicited measurable effects at the intestinal level, whereas corresponding hepatic improvements were still not detectable, particularly under sustained dietary challenge, thereby positioning the intestine and the gut microbiota as the earliest responsive targets of these interventions.

Gut microbiota composition was markedly altered by WD feeding in our MASLD–fibrosis model, with shifts in several taxa previously implicated in MASLD and liver fibrosis [23,52,53]. Melatonin and *A. muciniphila* administration produced modest but directionally consistent effects on gut microbiota composition, partially counteracting WD-induced disturbances, attenuating the increase in harmful taxa and promoting the enrichment of beneficial ones.

Among the genera enriched in the WD group, *Blautia* and *Alistipes* stood out for their consistent associations with adverse metabolic and hepatic outcomes. *Blautia* abundance correlated positively with serum cholesterol, ALT and *Timp1* expression, in line with previous studies linking this genus to dyslipidaemia [54,55] and hepatocellular injury [56]. *Alistipes* also showed positive correlations with cholesterol and *Timp1*, reinforcing its potential deleterious role in advanced stages of disease [57]. Neither melatonin nor *A. muciniphila* significantly reduced their abundance, which could indicate that these taxa may represent resilient features of WD-induced dysbiosis, although a non-significant trend towards lower *Alistipes* levels was observed with melatonin and towards lower *Blautia* with *A. muciniphila*. In addition, *Faecalibaculum* was also increased, consistent with associations with MASLD pathogenesis [58], obesity, gut inflammation, endotoxaemia and oxidative stress [59]. Its negative correlations with several dicarboxylic acids and mevalonate pathway intermediates suggest links with altered lipid [60] and cholesterol metabolism [61], supporting its contribution to metabolic and oxidative stress-related processes [62].

Both *Intestinimonas* and *Lachnoclostridium* were increased in the WD group and positively associated with cholesterol, glucose and metabolites such as hydroferulic acid, tromethamine and N-formylmethionine, the latter a pro-inflammatory bacterial peptide, reinforcing their reported involvement in metabolic and immune dysregulation [63,64]. Notably, *Intestinimonas* tended to decrease under the combined treatment, which could be beneficial given the links of these genera to dyslipidaemia, impaired glucose metabolism, oxidative stress and inflammation [65,66].

Other genera with potential harmful effects also showed increased abundance in the liver fibrosis model. *Lactococcus* and *Parasutterella* were enriched in WD-fed groups and showed negative associations with dicarboxylic acids and other metabolites linked to lipid metabolism and oxidative stress. While previous studies reported a reduction of *Lactococcus* after *A. muciniphila* [67] or antioxidant treatments [23,68], in the liver fibrosis model neither melatonin nor *A. muciniphila* significantly modified its abundance. *Tuzzerella*, another genus associated with metabolic stress, also remained elevated despite interventions. It displayed negative correlations with metabolites related to redox balance and energy metabolism, including antioxidants and TCA cycle intermediates, suggesting a potential involvement in oxidative stress and mitochondrial dysfunction.

Conversely, genera typically considered beneficial, such as *Muribaculum* and *Lactobacillus*, were reduced in WD-fed animals. *Lactobacillus* correlated negatively with inflammatory and fibrogenic gene expression. Given its well-described anti-inflammatory properties [69,70], its depletion in WD-fed animals is consistent with the pro-inflammatory environment of MASLD, where protective mechanisms against inflammation appear diminished. *Muribaculum* abundance correlated negatively with cholesterol and glucose and faecal concentrations of hydroferulic acid and N-formylmethionine, and positively with intestinal barrier gene expression (*Tjp1*) and faecal concentrations of 2-hydroxybutyric acid, consistent with its protective metabolic profile and its beneficial role in maintaining intestinal barrier integrity [71,72]. Aligning with reports of increased *Muribaculum* following probiotic [73] and antioxidant interventions [74], including studies with *A. muciniphila* [72], this genus showed a tendency towards restoration in the combined treatment group (WDMA).

Notably, melatonin and *A. muciniphila* did induce favourable shifts in other taxa. *Eisenbergiella*, which was enriched in the WDA and WDMA groups, correlated negatively with multiple metabolites linked to energy metabolism and oxidative stress and has been reported to be reduced in MASLD patients [75], suggesting a shift towards relative abundance values similar to the control group, possibly linked to a healthier profile.

In our study, *Odoribacter* abundance showed a downward trend across treatment groups. While previous reports have associated *Odoribacter* restoration after probiotic or antioxidant interventions with improved metabolic and inflammatory profiles [76,77], in our model, we observed other features of the gut microbiota composition and metabolism that suggest the initiation of an inflammatory remodelling process. Similarly, *Oscillibacter*, although frequently increased in MASLD models [78,79], was reduced in our WD-fed mice despite its positive correlation with serum triglyceride levels. Taken together, these findings reflect a context-dependent role of these genera in MASLD pathogenesis.

Regarding gut microbiota functionality, WD feeding led to increased levels of choline, muricholic acid and phosphocholine. Choline has been associated with steatosis, fibrosis and NASH risk [80,81], likely reflecting alterations in phospholipid metabolism and hepatic lipid export mechanisms. Muricholic acid elevation may reflect an altered bile acid profile consistent with MASLD-associated metabolic dysregulation. Importantly, tauro-β-muricholic acid, a farnesoid X receptor antagonist, tended to decrease in the WDA group, in line with previous studies [82] where *A. muciniphila* reduced this bile acid and improved disease features [83,84]. Phosphocholine, involved in very low-density lipoprotein-associated triglyceride synthesis, was also elevated, consistent with reports linking this metabolite to increased fibrosis severity [85].

Microbiota-derived tryptophan metabolites were particularly affected. Indole-3-acetic acid, consistently reduced in MASLD [86,87], showed a trend towards recovery in the WDA and WDMA groups, suggesting partial restoration of microbial metabolic activity and a shift to a more favourable environment, in agreement with its reported therapeutic effects, including reduced oxidative stress and inflammation, improved insulin resistance and lipid metabolism, and attenuation of steatosis and ballooning [86,87]. Indole-3-glyoxylic acid, another tryptophan-derived microbial metabolite with reported benefits on intestinal barrier integrity and inflammation control [88], was significantly decreased in our fibrosis model, consistent with the loss of a protective factor.

Isocitric acid, often elevated in MASLD, particularly in advanced disease stages [89,90], was significantly decreased in the WDA group, suggesting a treatment-related improvement. Sebacic acid, previously linked to insulin resistance and dyslipidaemia [91], was significantly reduced by the combined treatment (WDMA), and its inverse correlations with hepatic inflammation and fibrosis markers support a possible amelioration of disease severity.

Finally, phenylacetylglutamine, recognised as a hallmark metabolite of MASLD [92], was markedly increased in the liver fibrosis model. This amino acid–derived compound has been associated with adverse outcomes in cardiovascular and neurodegenerative diseases [93,94], while its reduction after BMI loss in obesity supports a link with metabolic improvement [95]. Interestingly, melatonin-treated animals (WDM and WDMA groups) exhibited elevated levels, a novel and unexpected finding not previously reported, which may point to complex or context-dependent effects of the treatment.

Our findings provide new insights into the multifactorial nature of MASLD and the complex interplay between gut microbiota, microbial- and host-derived metabolites and key molecular pathways involved in the pathogenesis of liver disease. The integrative analysis of gut microbiota composition, faecal metabolome, and gut and liver gene expression represents a noteworthy aspect of this study, as it enabled the identification of specific microbial and metabolic signatures associated with disease progression, which may serve as non-invasive biomarkers for disease monitoring or as therapeutic targets. Importantly, the administration of melatonin and *A. muciniphila*, alone or in combination, induced measurable changes in gut microbiota composition, faecal metabolome and intestinal gene expression, establishing microbial, metabolomic and intestinal signatures that could serve as a foundation for future studies exploring their potential as adjunctive therapies to modulate the gut-liver axis in MASLD. From a translational perspective, the combined administration of melatonin and *A. muciniphila* may offer several advantages over current therapeutic approaches. As a gut-targeted, low-cost and well-tolerated strategy, it could represent a valuable adjunct to lifestyle interventions and emerging pharmacological therapies, particularly in early or intermediate disease stages. By primarily modulating intestinal barrier function, microbiota composition and metabolic signalling, this approach may contribute to disease management with a favourable safety profile and minimal risk of adverse effects.

However, a number of limitations must be acknowledged. Melatonin and/or *A. muciniphila* produced minimal changes in hepatic and systemic parameters, likely reflecting the short treatment duration (four weeks) and the continued exposure to a Western diet, which may have limited their capacity to improve liver injury. Nevertheless, consistent trends in gut microbiota composition and function, as well as at the molecular level, including partial modulation of inflammatory and profibrogenic genes and restoration of barrier-related gene expression, indicate that these interventions elicit measurable effects in the gut and microbiota, providing a foundation for future adjunctive strategies targeting the gut–liver axis, particularly if applied earlier or under more favourable conditions.

Further research is warranted to elucidate the specific mechanisms through which *A. muciniphila* and melatonin exert their effects and to explore potential synergistic interactions. Extending the duration or initiating interventions earlier, as well as investigating other relevant tissues, such as adipose tissue or the gut–brain axis, may provide a more comprehensive understanding of their systemic impact. Ultimately, validation in human cohorts will be essential to confirm the identified microbial and metabolomic signatures and to assess the translational potential of these strategies as adjunctive approaches for MASLD management.

## 5. Conclusions

In conclusion, our results indicate that melatonin and *A. muciniphila*, administered alone or in combination, profoundly reshape gut microbiota composition and functionality and modulate intestinal gene expression, whereas the effects on hepatic parameters reflected only initial changes. These findings provide new insights into the interactions between the gut microbiota, microbial and host-derived metabolites and the intestinal environment in MASLD. The integrative multi-omic approach allowed for the identification of microbial and metabolic signatures, offering a foundation for future studies exploring their potential as biomarkers or therapeutic targets. Although improvements in liver function and histology were limited under fibrotic conditions, the observed modulation of the gut–liver axis suggests that targeting the microbiota and intestinal environment may represent a promising starting point for adjunctive therapies. Further research should assess longer or earlier interventions, dose–response effects and combined strategies to maximise therapeutic efficacy and clarify the mechanisms underlying gut–liver crosstalk in MASLD.

## Data Availability

The original data presented in the study are openly available in Zenodo at https://doi.org/10.5281/zenodo.18428901.

## Supplementary Materials

The following supporting information can be downloaded at: https://www.mdpi.com/article/10.3390/antiox15030306/s1, Table S1: Probes used for mRNA expression analyses by RT-qPCR, Table S2: Biochemical determinations after 8 weeks of WD feeding; Figure S1: Haematoxylin and eosin-stained liver sections (Scale bar: 100 µm); Figure S2: Masson’s trichrome-stained liver sections (Scale bar: 100 µm); Figure S3: Assessment of the comparability of control subgroups (C, CM, CA and CMA) for the analysed parameters using one-way ANOVA; Figure S4: Analysis of gut microbiota α-diversity measured by the Shannon and Simpson indices; Figure S5: Correlation analysis. Heatmaps showing Spearman correlations between (A) serum biochemical parameters and faecal metabolites, (B) hepatic gene expression and bacterial genera, (C) hepatic gene expression and faecal metabolites, and (D) intestinal gene expression and bacterial genera. Each square represents Spearman’s correlation coefficient (q < 0.05). Red and blue cells represent positive and negative correlations, respectively. White crosses designate the level of significance: + q < 0.05; ++ q < 0.01; +++ q < 0.001.
