# Supplementary material for: Effects of Melatonin and Akkermansia muciniphila on the Gut-Liver Axis in a MASLD-Associated Liver Fibrosis Model: An Integrative Multi-Omic Approach
Source: Antioxidants (Basel). 2026 Feb 28;15(3):306. doi: 10.3390/antiox15030306 (PMC13023461; doi:10.3390/antiox15030306)
Supplement: Supplementary file 1 [file antioxidants-15-00306-s001.zip › antioxidants-4160485-supplementary/Supplementary material.pdf]

Effects of Melatonin and *Akkermansia muciniphila* on the Gut-Liver Axis in a MASLD-Associated Liver Fibrosis Model: An Integrative Multi-Omic Approach

Alba González-Robles[1], Beatriz San Miguel[1], Sara Román-Sagüillo[1], María Juárez-Fernández[1], José Luis Mauriz[1], Susana Martínez-Flórez[1], Esther Nistal[1], María Victoria García-Mediavilla[1†], Sonia Sánchez-Campos[1†]

[1]Instituto Universitario de Biomedicina (IBIOMED), Universidad de León, León, Spain.

[†]These authors contributed equally to this work.

Supplementary Tables and Figures

Table S1 2
Table S2 2
Figure S1 3
Figure S2 3
Figure S3 4
Figure S4 4
Figure S5 5

Table S1. Probes used for mRNA expression analyses by RT-qPCR.

Gene name	Symbol	Genbank	Assay ID	Dye
Actin alpha 2	*Acta2*	NM_007392.3	Mm00725412_s1	FAM™
Claudin 1	*Cdln1*	NM_016674.4	Mm01342184_m1	FAM™
Mucin 2	*Muc2*	NM_023566.3	Mm01276676_m1	FAM™
NLR family, pyrin domain containing 3	*Nrlp3*	NM_145827.3	Mm00840904_m1	FAM™
Tight junction protein 1	*Tjp1*	NM_001157046.1	Mm01320638_m1	FAM™
Tissue inhibitor of metalloproteinase 1	*Timp1*	NM_001044384.1	Mm01341361_m1	FAM™
Toll-like receptor 2	*Tlr-2*	NM_011905.3	Mm01213946_g1	FAM™
Toll-like receptor 4	*Tlr-4*	NM_021297.2	Mm00445273_m1	FAM™
Tumor necrosis factor	*Tnf*	NM_001278601.1	Mm00443258_m1	FAM™
Glyceraldehyde-3-phosphate dehydrogenase	*Gapdh*	NM_008084.2	4352339E	VIC™

Table S2. Biochemical determinations after 8 weeks of WD feeding.

	C	WD
ALT (U/L)	66.19 ± 7.95	102.3 ± 8.63 **
AST (U/L)	78.98 ± 8.75	112.48 ± 7.38 **
Fasting blood glucose (mg/dL)	226.12 ± 5.93	272.43 ± 7.38 ****
Cholesterol (mg/dL)	65.88 ± 2.04	153.84 ± 5.23 ****

Values are represented as mean ± standard error of the mean (SEM). ** $p < 0.01$ vs. C; ****$p < 0.0001$ vs. C. ALT, alanine aminotransferase; AST, aspartate aminotransferase.

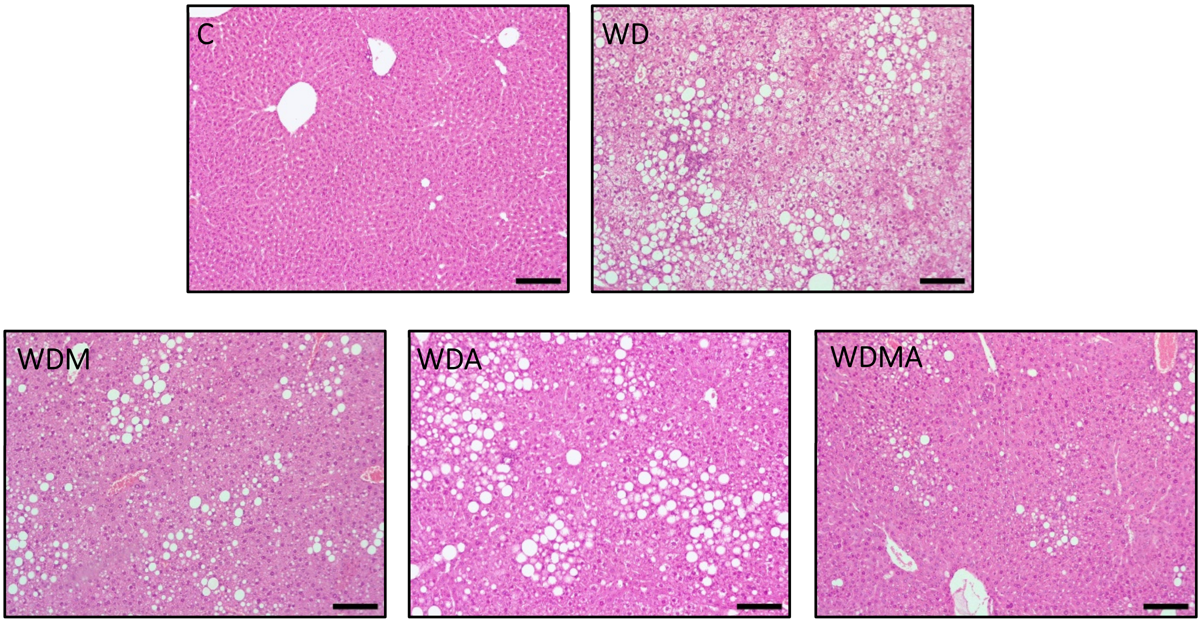

Figure S1. Haematoxylin and eosin-stained liver sections (Scale bar: 100 µm).

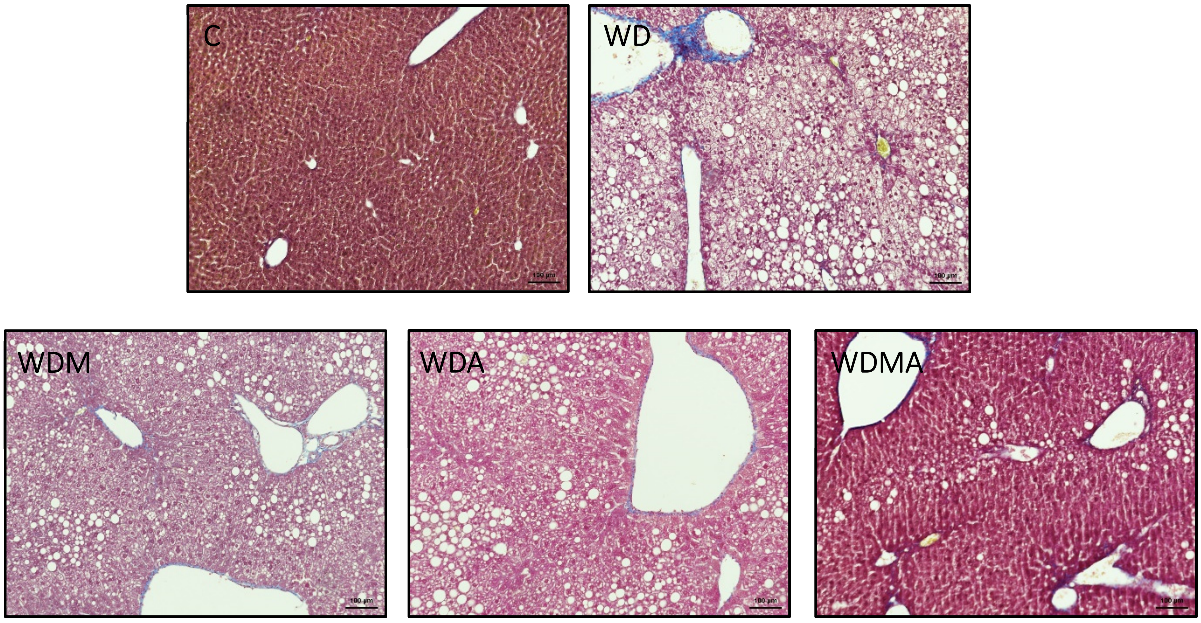

Figure S2. Masson's trichrome-stained liver sections (Scale bar: 100 µm).

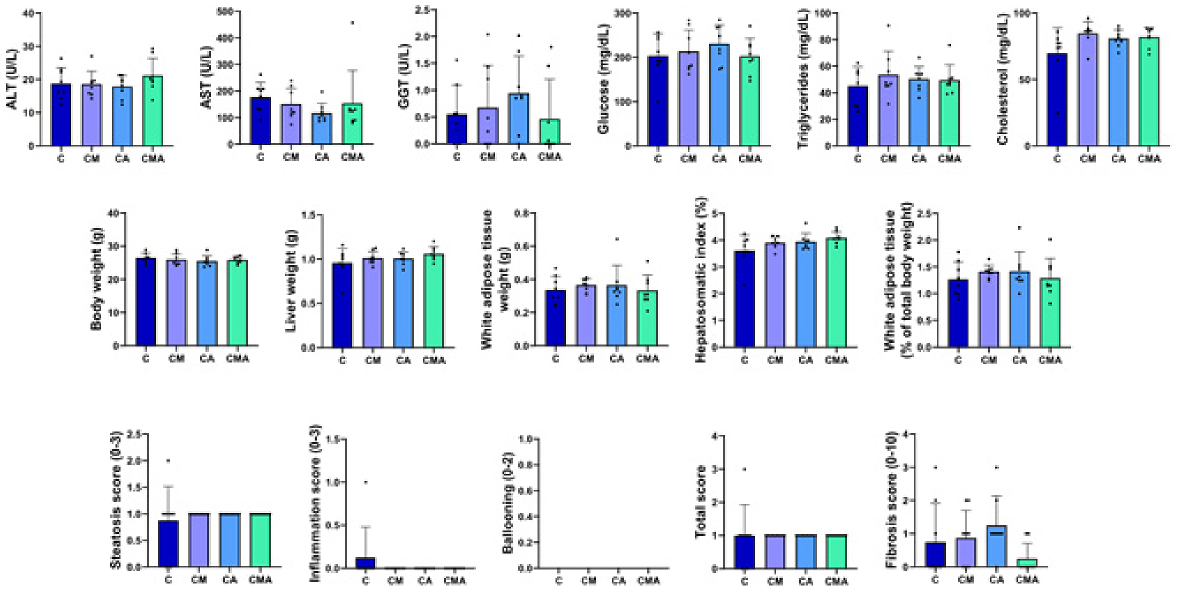

Figure S3. Assessment of the comparability of control subgroups (C, CM, CA and CMA) for the analysed parameters using one-way ANOVA.

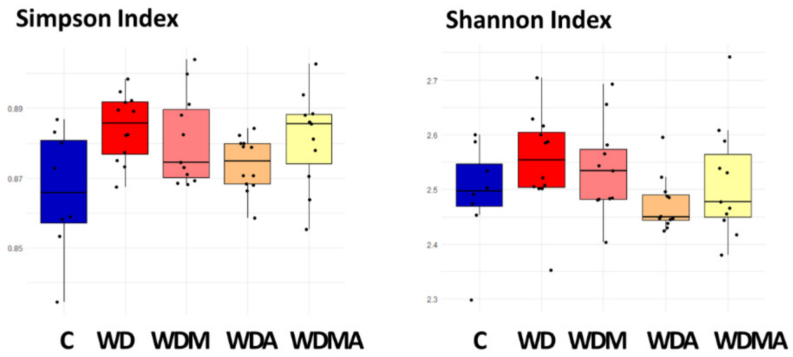

Figure S4. Analysis of gut microbiota α-diversity measured by the Shannon and Simpson indices.

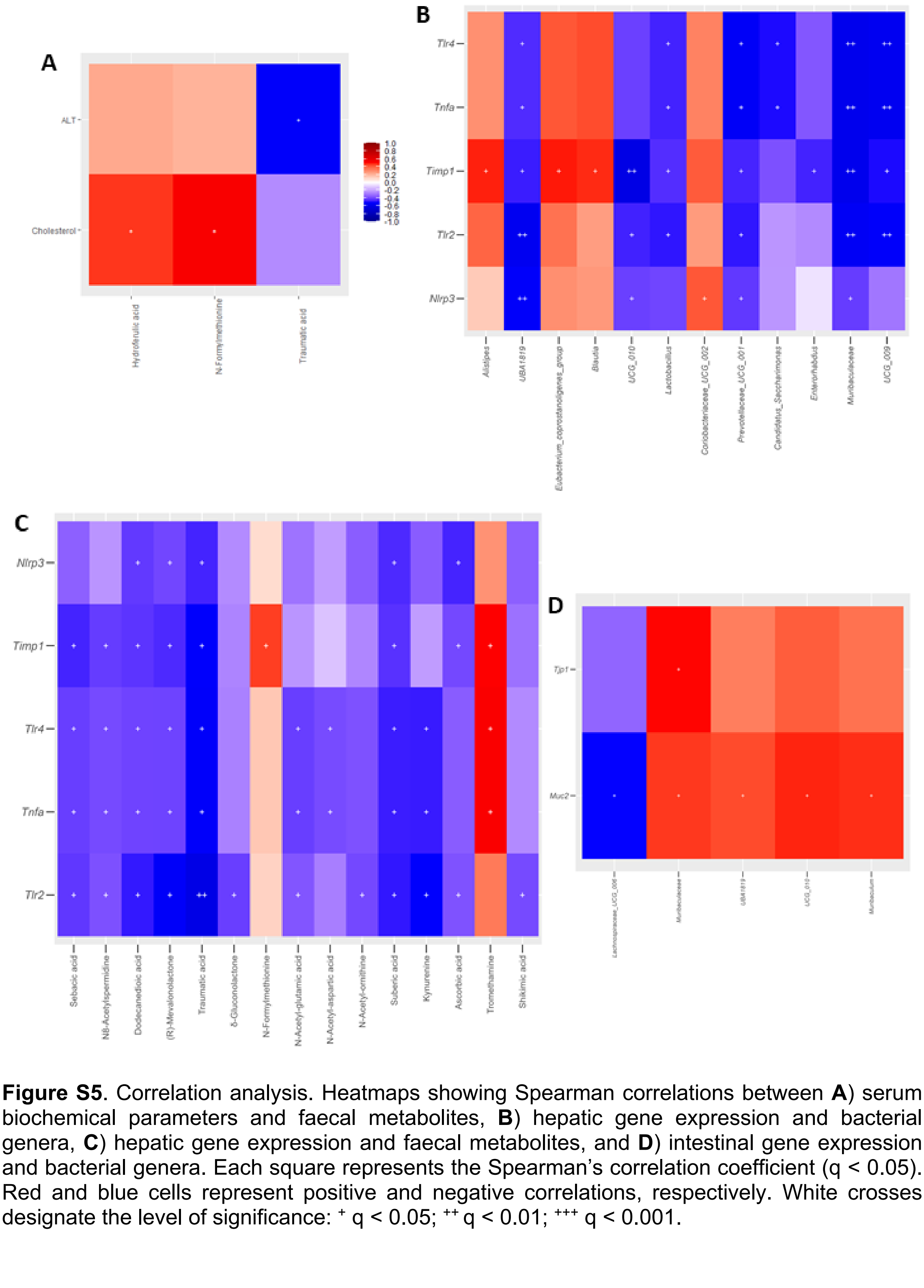

Figure S5. Correlation analysis. Heatmaps showing Spearman correlations between **A**) serum biochemical parameters and faecal metabolites, **B**) hepatic gene expression and bacterial genera, **C**) hepatic gene expression and faecal metabolites, and **D**) intestinal gene expression and bacterial genera. Each square represents the Spearman's correlation coefficient ($q < 0.05$). Red and blue cells represent positive and negative correlations, respectively. White crosses designate the level of significance: $^{+}$ $q < 0.05$; $^{++}$ $q < 0.01$; $^{+++}$ $q < 0.001$.
